# An improved Four-Russians method and sparsified Four-Russians algorithm for RNA folding

**DOI:** 10.1186/s13015-016-0081-9

**Published:** 2016-08-05

**Authors:** Yelena Frid, Dan Gusfield

**Affiliations:** Department of Computer Science, UC Davis, One Shields Avenue, Davis, CA USA

**Keywords:** RNA folding, Single sequence folding, RNA secondary structure, Secondary structure prediction, Four-Russians, Sparsification

## Abstract

**Background:**

The basic RNA secondary structure prediction problem or single sequence folding problem (SSF) was solved 35 years ago by a now well-known $O(n^3)$-time dynamic programming method. Recently three methodologies—Valiant, Four-Russians, and Sparsification—have been applied to speedup RNA secondary structure prediction. The sparsification method exploits two properties of the input: the number of subsequence *Z* with the endpoints belonging to the optimal folding set and the maximum number base-pairs *L*. These sparsity properties satisfy $0 \le L \le n / 2$ and $n \le Z \le n^2 / 2$, and the method reduces the algorithmic running time to *O*(*LZ*). While the Four-Russians method utilizes tabling partial results.

**Results:**

In this paper, we explore three different algorithmic speedups. We first expand the reformulate the single sequence folding Four-Russians $\Theta \left(\frac{n^3}{\log ^2 n}\right)$-time algorithm, to utilize an *on-demand* lookup table. Second, we create a framework that combines the fastest Sparsification and new fastest on-demand Four-Russians methods. This combined method has worst-case running time of $O(\tilde{L}\tilde{Z})$, where $\frac{{L}}{\log n} \le \tilde{L}\le min\left({L},\frac{n}{\log n}\right)$ and $\frac{{Z}}{\log n}\le \tilde{Z} \le min\left({Z},\frac{n^2}{\log n}\right)$. Third we update the Four-Russians formulation to achieve an on-demand $O( n^2/ \log ^2n )$-time parallel algorithm. This then leads to an asymptotic speedup of $O(\tilde{L}\tilde{Z_j})$ where $\frac{{Z_j}}{\log n}\le \tilde{Z_j} \le min\left({Z_j},\frac{n}{\log n}\right)$ and $Z_j$ the number of subsequence with the endpoint *j* belonging to the optimal folding set.

**Conclusions:**

The on-demand formulation not only removes all extraneous computation and allows us to incorporate more realistic scoring schemes, but leads us to take advantage of the sparsity properties. Through asymptotic analysis and empirical testing on the base-pair maximization variant and a more biologically informative scoring scheme, we show that this Sparse Four-Russians framework is able to achieve a speedup on every problem instance, that is asymptotically never worse, and empirically better than achieved by the minimum of the two methods alone.

## Background

Non-coding RNA (ncRNA) affects many aspects of gene expression, regulation of epigenetic processes, transcription, splicing, and translation  [14]. It has been observed that in eukaryotic genomes the ncRNA function is more clearly understood from the structure of the molecule, than from sequence alone. While there have been advances in methods that provide structure experimentally, the need for computational prediction has grown as the gap between sequence availability and structure has widened. In general, RNA folding is a hierarchical process in which tertiary structure folds on top of thermodynamically optimal^1^ secondary structure, secondary structure is a key component of structure prediction [14].

Efficient $O(n^3)$-time dynamic programming algorithms were developed more than thirty years ago to find non-crossing secondary structure of a single RNA molecule with *n* bases  [22, 23, 27, 29, 38, 39]. We call this basic folding or single sequence folding (SSF) problem. In addition, McCaskill [19] created an $O(n^3)$-time algorithm for the *partition function* for RNA secondary structure. Based on these algorithms, software has been developed and widely used  [15, 16, 25, 36, 37]. Probabilistic methods, employing Stochastic context-free grammar (SFCG), were also developed to solve the basic folding problem  [7, 8].

The accuracy of all these methods is based on the parameters given by the scoring function. Thermodynamic parameters [17, 18, 28, 33] and statistical parameters  [6, 7], or a combination of the two [2, 13] are currently employed.

The Valiant  [1, 34], Sparsification  [4, 30], and the Four-Russians (FR)  [9, 24] methods where previously applied to improve on the computation time for secondary structure prediction. For SSF, the Valiant method achieves the asymptotic time bound of $O\left(\frac{n^3}{2^{\Omega {\log (n)}}}\right)$ by incorporating the current fastest min/max-plus matrix multiplication algorithm  [32, 34]. The Four-Russians method was applied to single sequence [10, 24], cofolding [11] and pseudoknotted [12] folding problems. The Sparsification method, was developed to improve computation time in practice for a family of RNA folding problems, while retaining the optimal solution matrix [4, 20, 21, 26, 30, 35].

## Methods

In this paper, we combine the Four-Russians method  [24] and the Sparsification method  [4]. While the former method reduces the algorithm’s asymptotic running time to $\Theta \left(\frac{n^3}{\log ^2 n}\right)$, the latter eliminates many redundant computations. To combine these methods, we use an *on-demand* tabulation (instead of a preprocessing approach which is typically applied in FR algorithms), removing any redundant computation and guaranteeing the combined method is at least as fast as each individual method, and in certain cases even faster. First, we reformulate SSF Four-Russians $\Theta \left(\frac{n^3}{\log ^2 n}\right)$-time algorithm [24] to utilizes *on-demand* lookup table creation. Second, we combine the fastest Sparsification and Four-Russians SSF speedup methods. The Sparse Four Russians speedup presented here leads to a practical and asymptotically fastest combinatorial algorithm (even in the worst-case). The new algorithm has an $O(\tilde{L}\tilde{Z})$ run time where $\frac{{LZ}}{\log ^2 n}\le \tilde{L}\tilde{Z} \le \min \left( \frac{n^3}{\log ^2 n}, {LZ}\right)$. In practice, when accounting for every comparison operation the Sparse Four Russians outperforms both the Four-Russians and Sparsification methods. Third, we extended the Four-Russian SSF algorithm to be computed in $O(n^2/\log ^2n)$-time. The simulated results for this formulation and *O*(*n*) processors achieve a practice speedup on the number of comparison operations performed.

## Results

### Problem definition and basic algorithm

Let $s = s_0 s_1 \ldots s_{n-1}$ be an RNA string of length *n* over the four-letter alphabet $\Sigma = \{A,U,C,G\}$, such that $s_i \in \Sigma$ for $0 \le i < n$. Let $\varvec{ s_{i,j}}$ denote the substring $s_i s_{i+1} \ldots s_{j-1}$. We note that for simplicity of exposition substring $s_{i,j}$ does not contain the nucleotide *j*. A *folding* (or a *secondary structure*) of *s* is a set *M* of position pairs (*k*, *l*), such that: (1) $0 \le k< l < n$; (2) and there are no two different pairs $(k,l),(k', l') \in M$ such that $k \le k' \le l \le l'$ (i.e. each position participates in at most one pair, and the pairs are non-crossing).

Let $\beta (i,j)$ return a score associated with position pair (*i*, *j*). Let $\varvec{L}(s, M)$ be the score associated with a folding *M* of RNA string *s*, and let *L*(*s*) be the maximum score $\varvec{L}(s, M)$ over all foldings *M* of *s*. The **RNA Folding** or SSF problem is: given an RNA string *s*, compute *L*(*s*), and find an optimal folding *M* such that $L(s,M)=L(s)$. In this work, we assume the following simple scoring scheme:

$$\begin{aligned} L(s, M) = \sum _{(i,j) \in M} {\beta (i,j)}, \end{aligned}$$

where $\beta (i,j) = 1$ if $(s_i, s_j) \in \{(A, U), (U, A), (C, G), (G, C)\}$, and $\beta (i,j) = 0$ otherwise. Richer scoring schemes allow more biologically significant information to be captured by the algorithm. However, the algorithms for solving the problem similar recurrences and other discrete scoring schemes may be accelerated in a similar way to what we present here.

For the folding *M* of $s_{i,j}$, an index $k \in (i,j)$ is called a *split point* in *M* if for every $(x, y) \in M$, either $y < k$ or $k\le x$. A folding *M* is called a *partitioned folding* (with respect to $s_{i,j}$) if there exists at least one split point; otherwise *M* is called a *co-terminus folding*. Let the matrix *L* be a matrix such that $L[i,j] = L(s_{i,j})$. In addition, let $\varvec{L^p[i,j]}$ be the maximum value of $L(s_{i,j}, M)$ taken over all partitioned foldings *M* of $s_{i,j}$. Similarly, let $\varvec{L^c[i,j]}$ be the maximum value of $L(s_{i,j}, M)$ taken over all co-terminus foldings *M* of $s_{i,j}$. Let $L[i, i] = L[i, i+1] = 0$. For all $j > i+1$, *L*[*i*, *j*] can be recursively computed as follows ([23]):

1 $$\begin{aligned} L[i, j] = \max (L^p[i, j], L^c[i, j]),\end{aligned}$$

2 $$\begin{aligned} L^p[i, j] = \max _{k \in (i,j)}(L[i, k] + L[k, j]),\end{aligned}$$

3 $$\begin{aligned} L^c[i, j] = L[i+1, j-1] + \beta (i,j-1). \end{aligned}$$

For completeness, when $j < i$, define $L[i,j] = L^p[i,j] = L^c[i,j] = -\infty$.

The above recurrence may be efficiently implemented using a dynamic programming (DP) algorithm. Essentially, the DP algorithm computes and maintains values of the form $L[i, j], L^p[i, j]$ and $L^c[i, j]$ for every $0 \le i \le j \le n$ in three $n+1 \times n+1$ matrices. The algorithm traverses the matrices in increasing column order index *j* from 1 to *n*. Within each column, the cell *L*[*k*, *j*] is computed in decreasing index order *k* from $j-1$ to 0. Once *L*[*k*, *j*] is computed, $L^p[i,j]$ is updated for all $i<k$ such that $L^p[i,j]=max(L^p[i,j],L[i,k]+L[k,j])$. The solution *L*(*s*, *M*) is stored in cell *L*[0, *n*]. Clearly, computing $L^p$ is the bottleneck of the computation, since for a given *i*, *j*, there may be $\Theta (n)$ split points to examine.

### Extending the notation and moving towards a vector by vector computation of *L*

For a matrix *A* and some integer intervals *I*, *J*, denote by *A*[*I*, *J*] the sub-matrix of *A* obtained by projecting it onto the row interval *I* and column interval *J*. When $I = [i]$ or $J = [j]$, we simplify the notation by writing *A*[*i*, *J*] or *A*[*I*, *j*].

#### **Definition 1**

For a set of integers *K*, define the notation $L^p_K[i,j]$, and the max-plus operation $\otimes$ as

$$\begin{aligned} L^p_K[i,j] \;\;= \;\; L[i, K] \otimes L[K, j]\;\;=\;\;\displaystyle {\max _{k \in K}{(L[i,k] + L[k,j])}} . \end{aligned}$$

For an interval $I = [i, i + 1, \ldots i']$, define $L^p_K[I, j]$ to be the vector such that

$$\begin{aligned} L^p_K[I, j] \;\;=\;\; L[I, K] \otimes L[K, j] \;\;= \;\; \left[ L^P_K[i,j]\; \text {for all }\;{i\in I}\right] \end{aligned}$$

**Fig. 1:**
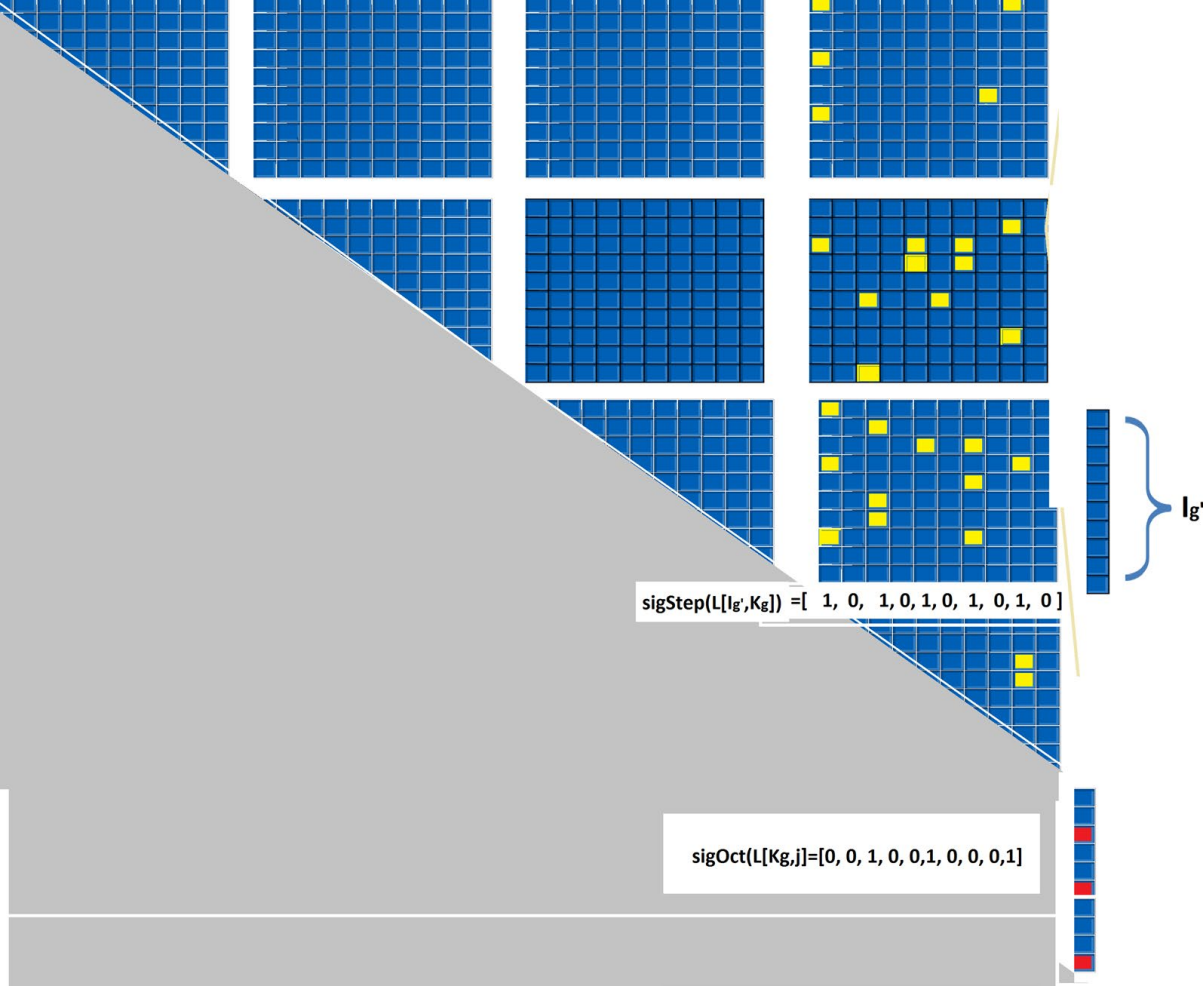
An example of how a solution matrix *L* is broken down into submatrices. Using the extended vector notation we can say that cell *L*[*i*, *j*] belongs to the vector $L[K_{g_i},j]$ as well as submatrix $L[I_{g_i},K_{g_j}]$. We partition the solution matrix *L* into $O(n^2/q)$ vectors of size *O*(*q*) and $O(n^2/q^2)$ submatrices, of size $O(q^2)$

We divide the solution matrix *L* in two ways: $q \times q$ submatrices (Fig. 1) and size *q* sub column vectors (the value of *q* will be determined later). Let $\varvec{K_g}$ be the *g*th interval such that $K_g=\{q \cdot g , q \cdot g+1, \ldots , q \cdot g+q-1\}$. We call these sets *Kgroups*, and use $K_g$ as the interval starting at index $g\cdot q$. For an index *i*, define $\varvec{g_i} = \left\lfloor \frac{i}{q}\right\rfloor$. It is clear that $i \in K_{g_i}$.

Similarly, we break up the row indices into groups of size *q*, denoted by $\varvec{I_g}$ where $I_g=\{k= q \cdot g , k+1, ...k+q-1\}$. (Clearly, row index set $I_g$ is equivalent to the Kgroup $K_g$. We only introduce this extra notation for simplicity of the exposition).

Given this notation $L^P[i,j]$ can be rewritten as maximization $L^p_{K_g}[i,j]$ values for all $K_g$ index Kgroups between *i* and *j*. However, in some cases, the indices $\{ i + 1, \ldots q\cdot g_{{i + 1}}  - 1\}$ do not form a full Kgroup $K_{g_i}$. Similarly indices $\{qg_j,qg_j+1, \ldots j-1\}$ do not form a full Kgroup $K_{g_j}$. Therefore, $L^P[i,j]$ can be computed by maximizing the full and non full Kgroups $K_g$. In Eq. 4 and the following sections we do not explicitly differentiate between full and non full groups.

4 $$\begin{aligned} \begin{array}{ll} L^p[i,j]&=\displaystyle \max _{g_i \le g \le g_j} L^p_{K_g}[i,j] \end{array} \end{aligned}$$

We extend the notation further, to compute the matrix $L^p$ not cell by cell but instead by vectors of size *q* corresponding to the $I_{g'}$ row sets, as follows.

5 $$\begin{aligned} \begin{array}{ll} L^p[I_{g'},j] =&\displaystyle \max _{g' \le g \le g_j} L^p_{K_g}[I_{g'},j]. \end{array} \end{aligned}$$

The DP algorithm can be updated to incorporate the extended notation. Within each column, compute the matrices in vectors of size *q*. Once $L[K_g,j]$ is computed it is used in computation of $L^p_{K_g}[I_{g'},j]$ for $g'<g$. When computing $L^p_{K_{g'}}[I_{g'},j]$ we follow Eq. 1–3 to complete the computation of cells $L[I_{g'},j]$.

### Sparsification of the SSF algorithm

**Fig. 2:**
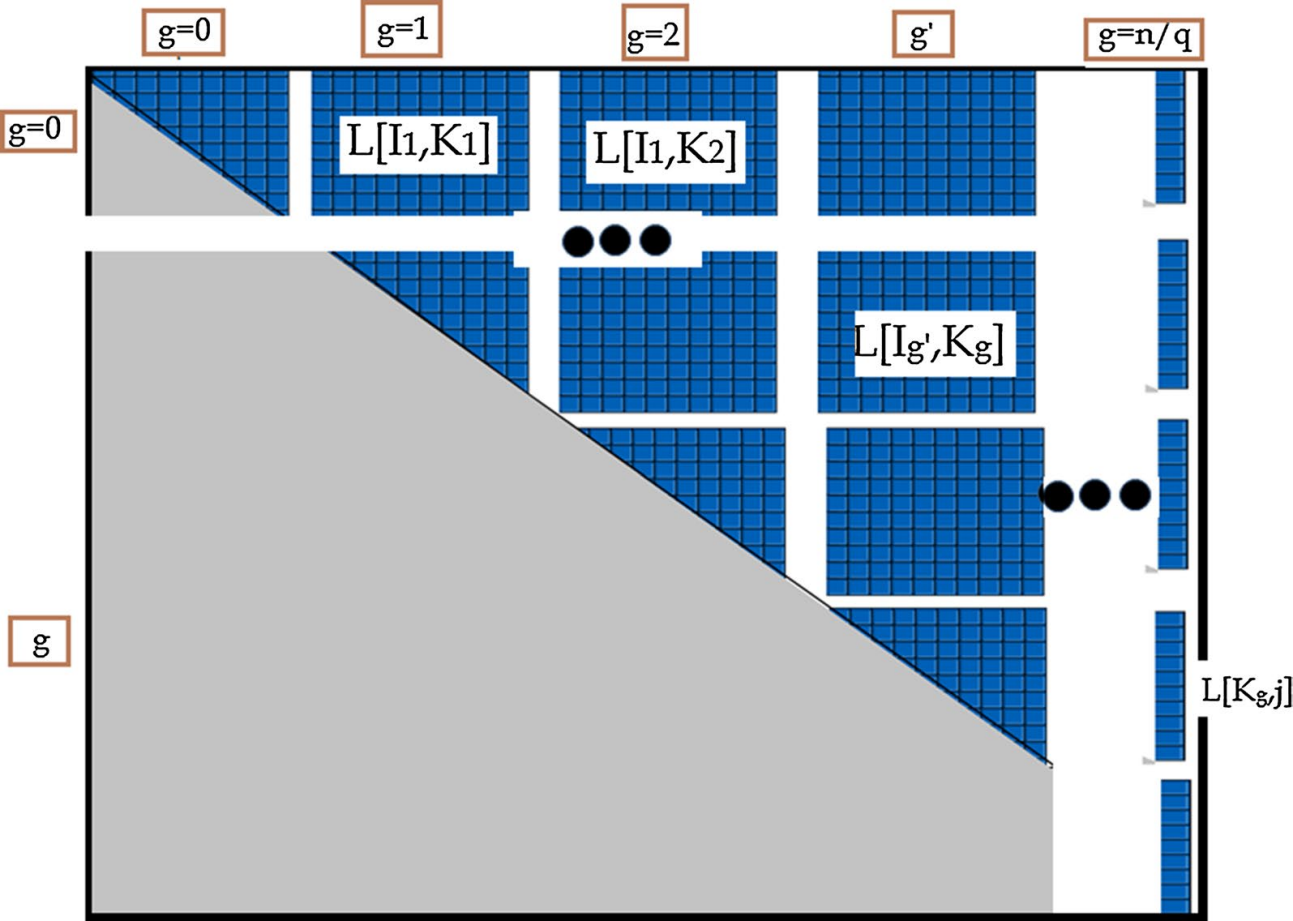
An sample examination to determine wether a submatrix and vectors are $STEP\!-\!OCT$. The yellow cells indicate *STEP* instances. The red cells indicate *OCT* instances. The $L[I_{g'},K_g]\otimes L[K_g,j]$ operation is only performed on submatrices with $sigSTEP \odot sigOCT >0$

The Sparsification method achieves a speedup by reducing the number of split points examined during the computation of $L^P[i,j]$. As Fig. 2 shows the focus of Sparsified Four Russians algorithm will narrow down only on those submatrices whose split points are  *step-oct* for a particular $i,j$ [4, 30].

#### OCT and STEP sub-instances of sequence *s*

Sub-instance $s_{i,j}$ is optimally co-terminus (*OCT*) if every optimal folding of $s_{i,j}$ is co-terminus. We introduce the extra notation below

if $L[i,j]=L^c[i,j]>L^p[i,j]$ then we say *L*[*i*, *j*] is *OCT*.

Sub-instance $s_{i,j}$ is *STEP*, if $L[i,j]>L[i+1,j]$ where $L[i,j]=L(s_{i,j})$ and $L[i+1,j]=L(s_{i+1,j})$. For ease of exposition we also say *L*[*i*, *j*] is *STEP* when $s_{i,j}$ is *STEP*. A *STEP* sub-instance $s_{i,j}$ implies that nucleotide *i* is paired in every optimal folding of $s_{i,j}$.

##### **Fact 1**

*For every sub-instance*$s_{i,j}$* with *$j>i$* there is an optimal split point *$k\in (i,j)$* such that either *$k=i+1$* orL*[*i*, *k*] is *STEP* and *L*[*k*, *j*] is *OCT*  [4].

**Notation:** For the index set $K=\{k,k+1, \ldots k'\}$ and column *j*, let $\varvec{K^{oct_j}}$ be the set of indices such that $K^{oct_j}\subset K$ and $\forall _{ k \in K^{oct_j}} \;\; L[k,j]$ is *OCT*. Given the row interval $I=\{i,i+1, \ldots i'\}$, let $I^{step_k}$ be the set of rows such that $I^{step_k} \subset I$, and for all ${i \in I^{step_k}}$*L*[*i*, *k*] is *STEP*.

We further define operation $\otimes _{step-oct}$ such that given $I=\{i,i+1,\ldots ,i'\}$ and $K=\{k,k+1,\ldots ,k'\}$, $L[I,K]\otimes _{step-oct} L[K,j]$ results in *A*[*I*, *j*] where $\displaystyle \forall _{i\in (I^{step_k} \cup I^{step_{k+1}}\cup \ldots I^{step_{k'}} )} A[i,j]$ is computed by the following procedure: 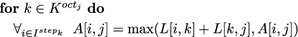


Using the operation $\otimes _{step-oct}$ and based on Fact 1. We reduce the time to compute $L^p[I_{g'},j]$ by considering a split-point *k* only if $k=i+1$ or *L*[*i*, *k*] is *STEP* and *L*[*k*, *j*] is *OCT* for $i\in I_{g'}$ and $k \in (i,j)$.

6 $$\begin{aligned} \begin{array}{ll} L^p[I_{g'},j]&=\displaystyle \max _{g' \le g \le g_j} L^p_{K_g}[I_{g'},j] =\displaystyle \max _{g' \le g \le g_j} L[I_{g'},K_g]\otimes _{{step-oct}} L[K_g,j]. \end{array} \end{aligned}$$

Note Eq. 6 does not explicitly show that for $L_{K_{g'}}^P[I_{g'},j]$ the split-point $i+1$ must be examined for every $i\in I_{g'}$.

*Asymptotic time bound of sparsified SSF* When computing matrix $L^p[i,j]$ we examine value *L*[*i*, *k*] only if *L*[*k*, *j*] is *OCT*. Let *Z*, be the total number of sub-instances in *s* or cells in matrix *L* that are *OCT*. Given that *L*[*k*, *j*] is *OCT*, $L^p[i,j]$ must examine the split point *k*, for all $i \in \{0,1, \ldots k\}$ such that *L*[*i*, *k*] is *STEP*. Let $\varvec{{L}}$ be the total number of *STEP* sub-instances in column *k*. More precisely ${L}=|\{0,1, \ldots k\}^{step_k}|$ (Creating the list of split-points that correspond to *STEP* incidence requires no additional computation time [4]). The total time to compute SSF when examining only *STEP*, *OCT* combinations (Sparsification method), is *O*(*LZ*). As shown in Backofen et al.  [4] *Z* is bounded by $Z\le n^2$ and *L* is bounded by ${L} \le \frac{n}{2}$. The overall asymptotic time bound of the Sparsification method is *O*(*LZ*) remains $O(n^3)$.

### On-demand Four Russians speedup

Presented here is an *on-demand* version of the $\Omega (\log ^2 n)$-time Four-Russians algorithm implied by Pinhas et al.  [24].

#### **Observation 1**

*The scores stored inL*[*k*, *j*] and $L[k+1,j]$* differ by the effect of adding only one more nucleotide (i.e., *$s_k$).* Therefore*, $L[k,j]-L[k+1,j]$* belongs to a finite set of differences*$\mathbb {D}$,* where*$\mathbb {D}$*is the set of scores created as the result of the scoring scheme*$\beta$.* The cardinality of the set of differences*, $D=|\mathbb {D}|$, is *O*(1)* when*$\beta$* is discrete. For the simple*$\beta$* scoring function (+1 for every permitted pair, and 0 otherwise), the set*$\mathbb {D}$* is equal to*$\{0,1\}$* and therefore*$|\mathbb {D}|=2$  [23].

Let $\vec {x} = [x_0, x_1, \ldots , x_{q-1}]$ be an integer vector of length *q*. We say that $\vec {x}$ is *D-discrete* if $\forall _{ l \in (0,q)} |x_{l-1} - x_{l}| \in \mathbb {D}$. We define the $\Delta$*-encoding* of 2-discrete vector $\vec {x}$ to be a pair of integers $(x_0, \Delta _{{x}})$ such that $x_0$ is the first element in $\vec {x}$ and $\Delta _{{x}}$ is the integer representation of the binary vector $[x_0-x_1, x_1-x_2, \ldots , x_{q-2} - x_{q-1}]$. Note that $0 \le \Delta _{{x}} < 2^{q-1}$. For simplicity, we will interchangeably use $\vec {x}$ to imply either $(x_0, \Delta _{x})$ or $[x_0, x_1, \ldots , x_{q-1}]$. Clearly, $\Delta$-*encoding* takes *O*(*q*) time to compute.

$\Delta$*-encoding vector operations:*

- Let $(x_0,\Delta _{\vec {x}})+ c= (x_0+c,\Delta _{\vec {x}})$ be equivalent to $\vec {x}+c =[x_0+c, x_1+c, \ldots , x_{q-1}+c]$.
- Let $B\otimes (x_0,\Delta _{x})$ be equivalent to $B\otimes \vec {x}$.
- Let $\max ((x_0,\Delta _x),(y_0,\Delta _y))$ be equivalent to $\max (\vec {x},\vec {y})$.

#### *MUL lookup table*

Based on Observation 1, any column vector in matrix *L* is 2-discrete. Given vector $L[K_g,j]$ and its $\Delta$*-encoding* ($x_0=L[gq,j]$, $\Delta _x= \Delta _{L[K_g,j]}$), it is clear that $\Delta _x \in [0,2^q-1]$.

##### **Fact 2**

$$L[I_{g'},K_g] \otimes L[K_g,j] \text { is equivalent to }L[I_{g'},K_g] \otimes (0,\Delta _{L[K_g,j]}) + L[gq,j]$$  [24].

*Let*$MUL_B[i]$* be a lookup table, where given a *$q \! \times \! q$* submatrix *$B=L[I_{g'},K_g]$* and*$i=\Delta _{L[K_g,j]}$,* the entry*$MUL_{L[I_{g'},K_g]}[\Delta _{L[K_g,j]}]=(y_0,\Delta _y)$* where *$\vec {y} =L[I_{g'},K_g] \otimes (0,\Delta _{L[K_g,j]})$.* We could reformulate the computation of*$L^p_{K_g}[I_{g'},j]$* to utilize theMUL lookup table*.

7 $$\begin{aligned} L^p_{K_g}[I_{g'},j]=L[I_{g'},K_g]\otimes L[K_g,j] = MUL_{L[I_{g'},K_g]}[\Delta _{L[K_g,j]}]+L[gq,j]. \end{aligned}$$

Equation 7, abstracts the detail that we still have to compute each referenced entry in the *MUL* lookup table. Each entry in the *MUL* lookup table is computed *on-demand* i.e. only when it corresponds to a required calculation. (This removes any extraneous calculation incurred when preprocessing all possible entries as in the typical Four-Russians implementation.) If entry $MUL_{L[I_{g'},K_g]}[\Delta _{L[K_g,j]}]$ does not exist we compute $L[I_{g'},K_g]\otimes (0,\Delta _{L[K_g,j]})$ directly in $O(q^2)$ time. If entry $MUL_{L[I_{g'},K_g]}[\Delta _{L[K_g,j]}]$ exists then the operation is *O*(1)-time lookup.

There are $O\left(\frac{n^2}{q^2}\right)$ submatrices within *L*. For each submatrix the maximum number of entries we compute for lookup table *MUL* is $2^{q-1}$. In total, the asymptotic time bound to populate lookup table *MUL* is $O\left(\frac{n^2}{q^2}\cdot 2^{q-1}\cdot q^2)=O(n^2 \cdot 2^q\right)$.

#### **MAX lookup table**

Let the *max* of two 2-discrete *q*-size vectors $\vec {v}$ and $\vec {w}$, denoted $max(\vec {v},\vec {w})$, result in a *q*-size vector $\vec {z}$, where $\forall _{0\le k < q}\, z_k=\max (v_k,w_k)$. Without loss of generality, let $w_0 \ge v_0$. Comparing the first element in each vector there are two possibilities either (1) $w_0-v_0>q-1$ or (2) $w_0-v_0\le q-1$. In the first case, ($w_0-v_0>q-1$), it is clear that $\max (\vec {v},\vec {w})$ is equal to $\vec {w}$. In the second case, we make use of the following fact  [24].

##### **Fact 3**

*Given two vectors*$(w_0,\Delta _w)$ and $(v_0,\Delta _v)$, if $w_0-v_0\le q-1$ then $\max (\vec {v},\vec {w})= \max \left( (0,\Delta _v),(w_0-v_0,\Delta _w)\right) +v_0$.

Lets define lookup table *MAX* such that entry

$MAX[i,i',h]=\max \left( (0,i),(h,i')\right)$. Hence, we reformulate Fact 3. to incorporate the *MAX* lookup table:

$$\begin{aligned} \max (\vec {v},\vec {w})=MAX[\Delta {v_0},\Delta {w_0},(w_0-v_0)]+v_0 \end{aligned}$$

We summarize these results in the function $\Delta$ max:

***Function***$$\Delta $$ max : : 
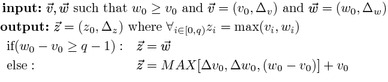


In Eq. 8, below, we integrate the vector comparison function $\Delta \max$. Each vector $L^p[I_{g'},j]$ is computed by maximizing over *O*(*n* / *q*) vectors. We will compute the lookup table *MAXon-demand* for every entry that does not exist an *O*(*q*). Clearly the lookup table $M\!A\!X$ will contain at most $2^{(q-1)}\cdot 2^{(q-1)}\cdot q$ for all entries. In worst case, the lookup table *MAX* computes in $O(2^{q^2}q)$ time.

8 $$\begin{aligned} L^p[I_{g'},j] = \varvec{\Delta }\!\!\!\!\max _{g' \le g \le g_j}\left( MUL_{L[I_{g'},K_g]} \left[ \Delta _{L[K_g,j]} \right] +L[gq,j] \right) \end{aligned}$$

The matrix $L^p$ and hence *L* is solved by a total of $O\left(\frac{n^2}{q}\right)$ computations of Eq. 8. In total, given lookup table *MUL* and $M\!A\!X$, the time to compute the Four-Russians SSF is $O\left(\underbrace{\frac{n^3}{q^2}}_{computation}+\underbrace{{2^{2q}}q+{n^2}{2^q}}_{\text {{ on-demand} lookup table }}\right)$.

Setting $q=\epsilon \log n$, where $\epsilon \in (0,.5)$  [31], the total computation time is equal to $\Theta (\frac{n^3}{\log ^2 n})$, which achieves a speedup by a factor of $\Omega {(\log ^2 n)}$, compared to the original $O(n^3)$-time solution method.

#### Extending to *D*-discrete vectors

We define the $\Delta$*-encoding* of *D*-discrete vector $\vec {x}$ to be a pair of integers $(x_0, \Delta _{{x}})$ such that $x_0$ is the first element in $\vec {x}$ and $\Delta _{{x}}$ is the integer representation in base 10 of the vector $[x_0-x_1, x_1-x_2, \ldots , x_{q-2} - x_{q-1}]$ in base D where $x_0$ is the most significant integer. Note that $0 \le \Delta _{{x}} < D^{q-1}$. As a result for a more complicated scoring scheme *B* we could apply the Four-Russians speedup by augmenting the *encode*, and *decode* functions as well as the $\Delta \max$ algorithm. 
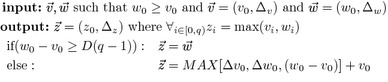


This would result in a total asymptotic time for Four-Russians SSF where $|D|>2$ of

$$\begin{aligned} O\left(\underbrace{\frac{n^3}{q^2}}_{computation}+\underbrace{{D^{2q}}q+{n^2}{D^q}}_{\text {{ on-demand} lookup table }}\right). \end{aligned}$$

Setting $q=\epsilon \log _D n$, where $\epsilon \in (0,.5)$  [31], the total computation time is equal to $\Theta \left(\frac{n^3}{\log ^2 n}\right)$, which achieves a speedup by a factor of $\Omega {(\log ^2 n)}$, compared to the original $O(n^3)$-time solution method.

## Sparse Four-Russian method

With the Four-Russians method, a speedup is gained by reducing *q* split point index comparisons for *q* subsequences to a single *O*(1) time lookup. The Sparsification method reduces the comparison to only those indices which correspond to *STEP*–*OCT* folds.

### *STEP–OCT* condition for sets of split points

In this section, we achieve a Sparsified Four-Russian speedup for the computation of the $L^p$ matrix. As in the Four Russians method, we will conceptually break up the solution matrix *L* in two ways: in $q \times q$ size submatrices, and *q* size subcolumn vectors. The submatrices are indexed by $g'$ and *g* such that the corresponding submatrix is $L[I_{g'},K_g]$ . The subcolumn vectors are indexed by *g* and *j* , such that the corresponding subcolumn vector is $L[K_g,j]$.

We augment the Four-Russians SSF to reduce the number of entries, and lookups into the *MUL* table. If and only if, the matrix $L[I_{g'},K_g]$ contains at least one cell *L*[*i*, *k*] that is *STEP* and within vector $L[K_g,j]$ the cell *L*[*k*, *j*] is *OCT* we will lookup $MUL_{L[I_{g'},K_g]}[\Delta _{L[K_g,j]}]$. If such an entry does not exist we will compute $L[I_{g'},K_g]\otimes (0,\Delta _{L[K_g,j]})$ and store the result into lookup table *MUL*.

The following notation will be used to help determine if a split point Kgroup should be examined in the computation.

#### *OCT subcolumn vector*

 Given the vector $L[K_g,j]$ let $\vec {m}$ be a *q* size binary vector such that $\forall _{0\le x \le q-1} m[x]=1$ if $L[gq+x,j]$ is *OCT*. Let the *sigOct* of the vector $L[K_g,j]$, written $sigOct(L[K_g,j])$, be equal to *m* the integer representation of the binary vector $\vec {m}$. Clearly $0 \le m < 2^q$, and if and compute the dot product in$m>0$ then $L[K_g,j]$ contains at least one *OCT* instance. Let $O(\tilde{Z})$ be the total number of subcolumn vectors which contain an instance that is *OCT*. Clearly, $\frac{{Z}}{q} \le \tilde{Z} \le \min \left(\frac{n^2}{q},Z\right)$.

#### *STEP submatrix*

 Given the submatrix $$L[I_{g'},K_g]$$, let $$\vec {m'}$$ be a *q* size binary vector such that $$\forall _{ x\in [0,q) }m'[x]=1$$ if $$\exists _{0 \le i \le q-1}$$$$L[qg'+i,qg+x]$$ is *STEP*. Let *sigStep* of a submatrix, written $$sigStep(L[I_{g'},K_g])$$, be equal to $$m'$$ the integer representation of the binary vector $$\vec {m'}$$. Clearly $$0\le m' < 2^q$$. Let $$\tilde{L}$$ be the total number of submatrices which contain an instance that is *STEP* within $$L[[0,n],K_g]$$. Clearly, $$ \frac{{L}}{q} \le \tilde{L} \le \min (\frac{n}{q},L) $$.

#### **Observation 2**

*Suppose that*, $s_{i,k}$* isSTEP*,* and integer*

$m' = sigStep(L[I_{g'},K_g])$* such that*$i \in I_{g'}$* (or*$I_{g'}=I_{g_i}$* ) and*$k \in K_g$* (or*$K_g=K_{g_k}$*). Then, the corresponding binary vector*$\vec {m'}$* must be set to 1 in positionx where x is an index such that*$k=qg+x$.* More precisely, ifL*[*i*, *k*]* isSTEP then*$m'[x]=1$* by the definition ofsigStep*.

#### **Observation 3**

*Suppose*$s_{k,j}$* isOCT, and suppose integer*

$m=sigOct(L[K_g,j])$* such that*$k \in K_g$.* Then, the corresponding binary vector*$\vec {m}$* must be set to 1 in positionx, wherex is an index such that *$k=qg+x$.* More precisely, if *$s_{k,j}$* isOCT thenm*[*x*] *= 1 by the definition ofsigOct*.

Given two binary vectors *v* and *w* the *dot product* of their integer representation is equal to a binary number *x* such that $x=v \odot w= v_0 \wedge w_0 \vee v_1 \wedge w_1 \vee ...\vee v_{q-1} \wedge w_q$ where $|v|=|w|=q-1$

#### **Theorem 1**

*For any subinstance *$s_{i,j}$* either*$i+1$* is the optimal split point, or there is an optimal split point *$k \in (i,j)$,* such that *$sigStep(L[I_{g_i},K_{g_k}]) \odot sigOct(L[K_{g_k},j])$* equals 1*.

#### Proof

Based on Fact 1 for any sub-instance $s_{i,j}$ there is an optimal split point *k* such that either $k=i+1$ or $s_{i,k}$ is *STEP* and $s_{k,j}$ is *OCT*. If $s_{i,k}$ is *STEP* and $s_{k,j}$ is *OCT* then *L*[*i*, *k*] is *STEP* and *L*[*k*, *j*] is *OCT*. The cell *L*[*i*, *k*] belongs to submatrix $L[I_{g_i},K_{g_k}]$ and the cell *L*[*k*, *j*] belongs to the vector $L[K_{g_k},j]$. Let *x* be an index such that $k=qg_k+x$. Let $\vec {m'}$ be a binary vector that corresponds to $sigStep(L[I_{g_i},K_{g_k}])$. Based on Observation 2, $m'[x]$ must equal 1. Let $\vec {m}$ be the binary vector that corresponds to $sigOct(L[K_{g_k},j])$. Based on Observation 3, *m*[*x*] equals 1. Therefore, $m[x]\wedge m'[x]=1$ and $sigStep(L[I_{g_i},K_g])\odot sigOct(L[K_g,j])= 1$. $\square$

**Notation:** The index *g* is *STEP*–*OCT* if given the set of rows $I_{g'}$ and the column *j* if $sigStep(\;L[I_{g'},K_g]\;) \varvec{\odot } sigOct(\;L[K_g,j]\;)=1$.

We can reformulate the computation of $L^p[I_{g'},j]$ by referencing the lookup table *MUL* only if *g* is *STEP*–*OCT*. This reduces the number of operations used in computing the bottleneck $L^P$ matrix.

9 $$\begin{aligned} L^p[I_{g'},j] = \Delta \!\!\!\!\!\!\!\!\!\!\!\displaystyle \max _{\begin{array}{c} g \text { is } S\!T\!E\!P\!-\!O\!C\!T \\ \text {where } g \in [g',g_j] \end{array}} \left( MUL_{L[I_{g'},K_g]} \left[ \Delta _{L[K_g,j]}\right] + L[gq,j]\right) \end{aligned}$$

We update the DP algorithm to only access the *MUL* lookup table for matrix and vector combinations that satisfy the property

$$sigStep(\;L[I_{g'},K_g]\;) \varvec{\odot } sigOct(\;L[K_g,j]\;)=1$$.

Let *G* be a lookup table, where give an index $g\in [0,n/q]$ and integer $m\in [0,2^q]$ the $G[g][m] \subset \{I_0,I_1,\ldots ,I_g\}$ is a set of row index intervals. Each index $I_{g'}$ within G[g][m] satisfies the following condition:

$$\begin{aligned} \text {if } I_{g'} \in G[g][m] \text { then } sigStep(L[I_{g'},K_g]) \varvec{\odot } m=1 . \end{aligned}$$

Lookup table *G* (updated *on-demand*) allows us to implement Eq. 9. As $L[K_g,j]$ is computed, the corresponding *SigOct* is also computed. Let $m=sigOct(L[K_g,j])$. By iterating through $I_{g'}\in G[g][m]$ set of row indices we access table *MUL* only when both of the following conditions hold at the same time: the submatrix $L[I_{g'},K_g]$ contains at least one cell *L*[*i*, *k*] where $s_{i,k}$ is *STEP* and within vector $L[K_g,j]$ the cell *L*[*k*, *j*] contains $s_{k,j}$ that is *OCT* (where $i\in I_{g'}$ and $k \in K_g$).

The Sparsified Four-Russian algorithm implements Eq. 9. The *complete* function will tabulate *STEP*, and *OCT* instances as well as *sigStep* and *sigOct* values. The *G*, *MUL* and *MAX* lookup tables will be computed *on-demand*. 
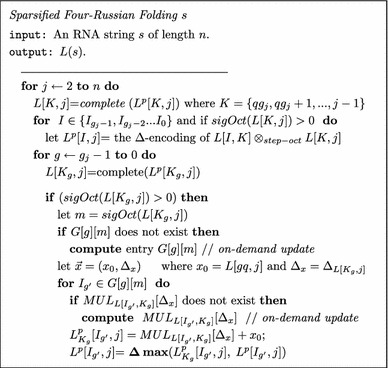

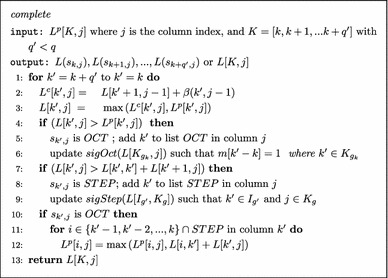

## Discussion

### Asymptotic analysis of sparsified Four-Russians.

We assume *O*(1)-time RAM access for $$\log (n)$$ bits. The calculation for column *j* can be broken down into $$L^P_{K=[qg_j,j)}[i,j]$$ and $$L^P_{K=[0,qg_j)}[i,j]$$ for all $$i<j$$. The computation of

$$L^P_{[qg_j,j)}[[0,n],j]$$ occurs when Kgroup $$K_{g_j}$$ is not full, and follows the Sparsification algorithm maximizing over * STEP*–*OCT* split points only. This reduces the comparisons made from $$O(n\cdot q)$$ to $$O({L}\tilde{q})$$ where $$\tilde{q}<q$$ is the total number *OCT* instances within the interval $$[qg_,j)$$. The computation of $$L^P_{[0,qg_j)}[[0,n],j]$$ employs Sparsified Four Russians speedup. The *MUL* table entries are created and references only for* STEP*–*OCT *submatrix vector combinations. This reduces the comparisons made to $$O(\tilde{L}\tilde{Z})$$.

The helper function *complete* is called $O(n^2/q)$ times for the entire algorithm. The *complete* function outer-loop iterates at most *O*(*q*) times updating the lists of *OCT* and *STEP* split points, as well as *sigOct* and *sigStep* values. Overall the *complete* function takes $O(q+\tilde{x})$ where $\tilde{x} \le q^2$ is the number of* STEP*–*OCT* instance combinations. The asymptotic runtime of the Sparsified Four-Russian algorithm is

$$\begin{aligned} O(\tilde{L}\tilde{Z})+O\left(\frac{n^2}{q}\cdot \tilde{x}\right)+O\left(\text {updating lookup tables on-demand}\right)=O(\tilde{L}\tilde{Z}) \end{aligned}$$

### Asymptotic analysis of on-demand lookup tables calculation

We compute the lookup tables *G*, *MUL*, and $M\!A\!X$*on-demand*. For each vector $L[K_g,j]$ containing an *OCT* instance (where $m=sigOct(L[K_g,j])$), if *G*[*g*][*m*] does not exist then we directly compute it. For the computation of a single entry into lookup table *G*, we iterate through $O(\tilde{L})$ submatrices and compute the dot product in *O*(*q*) time.^2^In total, an update is called to lookup table *G* at most $O(\tilde{C}=min(2^q,\tilde{Z}))$ times. The entire *G* lookup table on-demand computation takes $O(\text {on-demand} G)=O(\tilde{L}\tilde{C}\cdot q)$ or $\varvec{O(G)} \le O\left( \min (\tilde{L} 2^q,\tilde{L}\tilde{Z})\cdot q \right)\le O\left(min\left(\frac{n2^q}{q},\frac{{LZ}}{q}\right)\right)$.

For each vector containing an *OCT* instance if an entry doesn’t exist in the lookup table *MUL* it is computed on-demand. Each entry takes $O(\tilde{L}\cdot q^2)$ time to compute. There are $min(2^q,\tilde{Z)}$ such computation. In total, lookup table *MUL* takes $O(\tilde{L}q^2\cdot min(2^q,\tilde{Z}))$-time. Setting $q=\epsilon \log {n}$ where $\epsilon \in (0,.5)$ the asymptotic run-time for *on-demand* computation is $O(\tilde{L}\tilde{Z})$.

The entire algorithm takes $O(\tilde{L}\tilde{Z})$ where $\frac{{LZ}}{\log ^2 n}\le \tilde{L}\tilde{Z} \le \min \left(\frac{n^3}{\log ^2 n}, {LZ}\right)$.

### Empirical results

We tested 20 randomly generated sequences for each size $N=64,128,256,512$.

The empirical testing results are given not in seconds but in the number of operations including both lookup table creation and split-point comparisons. We do so to abstract from the effect compiler optimizations. Note that the testing does not account for memory access time, or extend the algorithm to $D>2$ scoring schemes (Table 1).

**Table 1:** Number of all comparisons computed

Size	$O(n^3)$	FR	SP	SFR
64	43,680	12,014	2733	1837
128	349,504	49,456	13,196	9982
256	2,796,160	346,692	79,544	41,393
512	22,500,863	5,746,853	650,691	503,425

For $N=128$ the Sparse Four-Russians(SFR) algorithm performs 25 % less comparisons than the Sparsified(SP) SSF algorithm and 80 % less comparison than the Four-Russians (FR) algorithm. In all test cases, the Sparse Four-Russians performed better than the minimum of either method alone.

### An $O(n^2/\log ^2(n))$ simple parallel Four-Russians RNA folding algorithm

Lets solve the recurrence relation (Eq. 1–3) in increasing index *j* order and then move up the column *j* computing one cell at a time in decreasing *i* order. Each cell *L*[*i*, *j*] is solved by calculating Eq. 1–3 for all $i<k\le j$.

Given this *j*, *i*, *k* order, let us reformulate the computation by moving up each column in *O*(*n*/*q*) *q*-size subcolumn vectors instead of *n* cells.

#### Utilizing *n* processors

Lets create a new process for each column *j*, creating *n* process in total. We can synchronously move up the matrix computing each column subvector such that on iteration *d* we compute $L[I_{g_j-d},j]$ for all $j \in (0,n)$. 
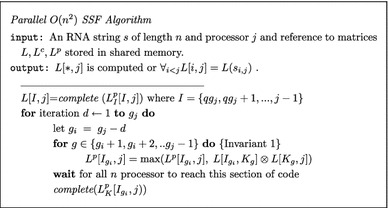


##### Invariant 1

Given $$g_i$$ and $$g_j$$$$\forall _{i\in I_{g_i}} \forall _{k \in K_g} L[i,k]=L(s_{i,k})$$. In other words, submatrix $$L[I_{g_i},K_g]$$ is computed. Similarly $$L[K_g,j]$$ is computed or $$\forall _{k \in K_g} L[k,j]=L(s_{k,j})$$.

Please note that the function *complete* assumes that $L^p_{\overline{K}}[I,j]$ is computed, where $\overline{K}=\{i,i+1, \ldots j-2,j-1\} - K$. 
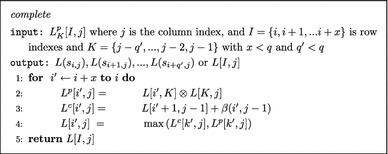


Replacing the $\max (L^p[I_{g_i},j], L[I_{g_i}, K_g])\otimes L[K_g,j])$ computation with lookups into *MUL* and *MAX* tables would reduces the run-time for finding the solution matrix *L* to $O(n^2/log^2n)$. As stated in "Extending to D-discrete vectors" section it is possible to create lookup tables on-demand and achieve a reduction in computation time of $\Omega (log^2 n)$ factor.

The preprocessing can also be achieve in parallel reducing the asymptotic cost form $O(n^3/\log ^2 n)$ to $O(n^2/\log ^2 n)$. If entry $MUL_{L[I_{g_i},K_g]}[\Delta _{L[K_g,j]}]$ does not exist we compute $L[I_{g_i},K_g]\otimes (0,\Delta _{L[K_g,j]})$ directly in $O(q^2)$.

There are $O\left(\frac{n^2}{q^2}\right)$ submatrices within *L*. For each submatrix the maximum number of entries we compute for lookup table *MUL* is $D^{q-1}$. However, in each iteration at worse *O*(*n*) of the entries are computed simultaneously. In total, the asymptotic time bound to populate lookup table *MUL* is $O\left(\displaystyle \frac{{\frac{n^2}{q^2}\cdot D^{q-1}\cdot q^2} }{n} \right)=O\left(\frac{n^2 \cdot D^q}{n}\right)=O(n \cdot D^q)$.

Based on Williams  [31] $O(D^q)$ is bound by $O(n/\log ^2 n)$ when setting $q=\epsilon \log n$. Hence, for the *MUL* lookup table the total asymptotic computation time is $O(n \cdot D^q)=O(n^2/\log ^2 n)$, For the *MAX* table similarly the serial computation of $O(D^{2q}q)$ total time is reduced by a factor of *n* in the parallel case. The total computation time for the *MAX* table is therefore $O(n/\log ^3 n)$. 
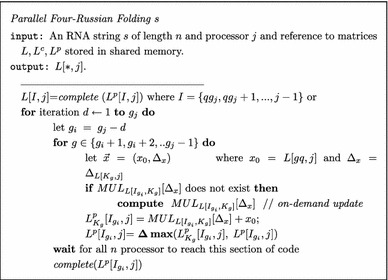


#### Parallel sparisified Four-Russians single sequence folding algorithm

Let $Z_x$ be the number of *OCT* cells in column *x*. Let $\forall _{x\in [0,n]}Z_j \ge Z_x$.

The parallel algorithm would take as long as would take as a it takes for the last processor to complete.

In order to extend the parallel Four-Russians single sequence folding algorithm to utilize the Sparsification speedup we will limit the call to *MUL* table only if $sigSTEP(L[I_{g_i},K_g]) \odot sigOCT(L[K_g,j]) >0$. As result given $Z_j$ the total time to compute for processor *j* is $O(\tilde{L}\tilde{Z_j})$ where $\frac{{Z_j}}{\log n}\le \tilde{Z_j} \le min \left({Z_j},\frac{n}{\log n}\right)$.

## Conclusion

This work combines the asymptotic speedup of Four-Russians with the very practical speedup of Sparsification. The on-demand formulation of the Four-Russians not only removes all extraneous computation. This approach allows the Four-Russians SSF to achieve a speedup in practice for realistic scoring schemes. This also leads us to take advantage of the sparsity properties. Through asymptotic analysis and empirical testing on the base-pair maximization variant and a more biologically informative scoring scheme, we show that the Sparse Four-Russians framework is able to achieve a speedup on every problem instance, that is asymptotically never worse, and empirically better than achieved by the minimum of the two methods alone. We also showed that through some re-organization we could apply the Four-Russians speedup to parallel algorithm and achieve and asymptotic time of $O(n^2/\log ^2 n)$. The algorithm created here can be implemented in CUDA to compute on multiprocessor GPUs. Because the algorithm allows for memory cell independence one can apply memory and cache optimization without affecting the algorithm. The utility in this framework lies not only on its ability to speedup single sequence folding but its ability to speedup the family of RNA folding problems for which both Sparsification and Four-Russians have bene applied separately.

Future work in this area would be to examine the ability to sparsify memory [3], as Four-Russians at worst case requires an additional factor of $2^{log(n)}$ in memory. Another open question is wether it is possible to apply the $\Omega (\log ^3 n)$ [5] speedup of boolean matrix multiplication to RNA folding.

## Footnotes

1 Or close to optimal.

2 Using some word tricks the dot product could be computed in *O*(1)-time.
